# Scanning Kelvin Probe Microscopy: Challenges and Perspectives towards Increased Application on Biomaterials and Biological Samples

**DOI:** 10.3390/ma11060951

**Published:** 2018-06-05

**Authors:** Marco Salerno, Silvia Dante

**Affiliations:** 1 Materials Characterization Facility, Istituto Italiano di Tecnologia, via Morego 30, 16163 Genova, Italy; marco.salerno@iit.it; 2 Department of Nanoscopy & NIC, Istituto Italiano di Tecnologia, via Morego 30, 16163 Genova, Italy

**Keywords:** surface potential, biomaterials, living cells substrates, electrical cues, biomolecules

## Abstract

We report and comment on the possible increase of application of scanning Kelvin probe microscopy (SKPM) for biomaterials, biological substrates, and biological samples. First, the fundamental concepts and the practical limitations of SKPM are presented, pointing out the difficulties in proper probe calibration. Then, the most relevant literature on the use of SKPM on biological substrates and samples is briefly reviewed. We report first about biocompatible surfaces used as substrates for subsequent biological applications, such as cultures of living cells. Then, we briefly review the SKPM measurements made on proteins, DNA, and similar biomolecular systems. Finally, some considerations about the perspectives for the use of SKPM in the field of life sciences are made. This work does not pretend to provide a comprehensive view of this emerging scenario, yet we believe that it is time to put these types of application of SKPM under focus, and to face the related challenges, such as measuring in liquid and quantitative comparison with other techniques for the electrical potential readout.

## 1. Introduction

Scanning Kelvin probe microscopy (SKPM) is a variation of atomic force microscopy (AFM) that makes it possible to map not only the morphology of solid sample surfaces, but also their electrical potential [1]. So far, SKPM has been applied mainly on semiconductor and metal systems, either to gain a deeper understanding of their electron transport properties in terms of work function, or to characterize optoelectronic devices, such as transistor junctions or heterostructures for laser cavities, or solar cells during operation [2]. However, room also exists for the successful application of this technique in the field of biophysics and biomedical surfaces [3]. In fact, the electrical surface charge is at the base of many biomolecular interactions [4], and complements the substrate chemical nature, morphology and stiffness as one of the major adhesion cues for living cells in culture [5,6].

In this work, without entering into a detailed analysis of the operating SKPM setup, we first discuss the meaning of the SKPM data and the practical limitations of the measurement technique; then we report about the use of SKPM on different types of samples of biological interest, including living cell substrates, based on the existing literature in the area and on our experience.

## 2. Considerations about SKPM Data

### 2.1. SKPM Image Contrast: Meaning and Limitations

**Figure 1:**
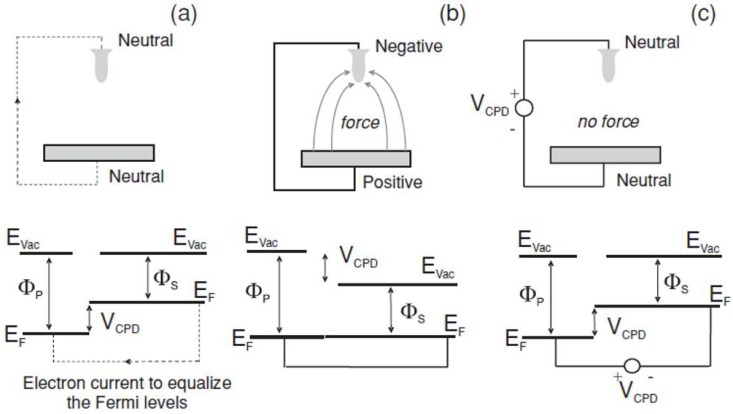
Operating principle of the Kelvin probe. In this picture probe (sample) is on top (bottom) in the sketches and on left (right) in the energy diagrams, respectively. Reproduced from Ref. [2] with permission; Copyright Springer 2007. (**a**) Before and (**b**) after making the electrical connection; (**c**) after proper bias.

When measuring locally on a fixed sample position, the Kelvin probe technique [7] generally allows the experimenter to read a potential difference arising from the combination of interacting probe and sample materials (assumed to behave as metals), as shown in the picture in Figure 1.

After alignment of the Fermi levels *E_F_* in the two conductors placed into contact (Figure 1b), the difference in work functions Φ (i.e., the energy required to extract one electron to the vacuum level) gives rise to a relative contact potential difference (CPD), emerging at the sample surface with respect to the probe, also called surface potential *V_sp_*. The latter name is conveniently easy to remember after the indexing initials of ‘surface potential’, as this by chance coincides with the initials of sample and probe, respectively, i.e., it is *V_sp_ = V_sample_ − V_probe_*. The following relation holds:

(1) $$\Phi_{probe}-\Phi_{sample}=sign\left(V_{sample}-V_{probe}\right)\;eV_{sp}$$

The sign in Equation (1) depends on the sign of the bias applied to the probe with respect to the sample in order to nullify the *V_sp_*. So the sign is plus when the probe is positively biased with respect to the sample (as in Figure 1c). It should be noted that this is not the case in all the experimental setups, as applies for instance in SKPM implementations by, e.g., a given AFM manufacturer [8] (where the sample is set to virtual ground and *V_probe_* > 0), whereas in those made by, e.g., another manufacturer [9], the opposite holds (i.e., *V_sample_ − V_probe_* > 0).

In SKPM, the moving probe and sample behave as the plates of a capacitor, with dielectric air in between, and the modulation of distance along the *z* axis gives rise to a changing force, with components of different frequencies: DC, AC at cantilever frequency modulation ω, and AC at 2ω. The dominating term is the one at ω frequency, which enters into a feedback system. The corresponding equations are nicely described in most AFM manuals for SKPM mode (see, e.g., Ref. [2,8,9]). Here we do not intend to repeat these technical considerations, but rather invite to reflect on the meaning of the SKPM measurement itself. To this end, one should restart from Equation (1), converting the sample material work function to the apparent electrical surface potential. In the following, it will be assumed that *V_probe_ > V_sample_*. In these conditions one has:

(2) $$\Phi_{tip}-\Phi_{sample}=eV_{sp}$$

Notice that we have replaced “probe” with “tip”, as in SKPM the probe is not a wide plate such as that in the original static Kelvin setup, but is a scanning probe with a terminal sharp tip, providing the resolution required for microscopy. When one further simplifies the expression by shortening the indices (from “tip” to “t” and from “sample” to “s”), and introduces the SKPM dependence on the local sample surface position during the scan, (*x*, *y*), Equation (2) becomes:

(3) $$\Phi_{t}-\Phi_{s}\left(x,y\right)=eV_{sp}\left(x,y\right)$$

This is actually the “golden” equation of SKPM, which is used at different steps of the measurement procedure. When it is necessary to gain information about the sample work function Φ*_s_*, Equation (3) has to be resolved for Φ*_s_*, but the Φ*_t_* has to be known, which, in turn, is determined by assuming the Φ*_s_* to be known, on a sample with given nominal properties (“test” sample), and Equation (3) has to be resolved first for Φ*_t_* on such a sample; a procedure known as “tip calibration”:

(4) $$\Phi_{t}=eV_{sp\;test}+\Phi_{s\;test}$$

In most cases, this test sample is highly oriented pyrolytic graphite (HOPG), which is atomically flat, i.e., morphologically featureless, with assumed Φ*_HOPG_* = 4.65 eV (see Ref. [10,11]); sometimes, gold with crystal orientation (111) obtained by clean flame annealing is also used, for which Φ*_Au_*_(111)_ = 5.31 eV [12].

It can be seen that in Equation (4), the local dependence on the tip position on the sample has been ruled out, in that, for best calibration conditions, the sample is also assumed to be uniform, such that *V_sp test_(x,y)* is constant, after Φ*_s test_(x,y)* was constant as well. This is more easily the case for HOPG, while for gold, several defects often appear, mainly at the corners and edges of (111) terraces, due to local burning, at least when homemade in the laboratory by flame annealing.

**Figure 2:**
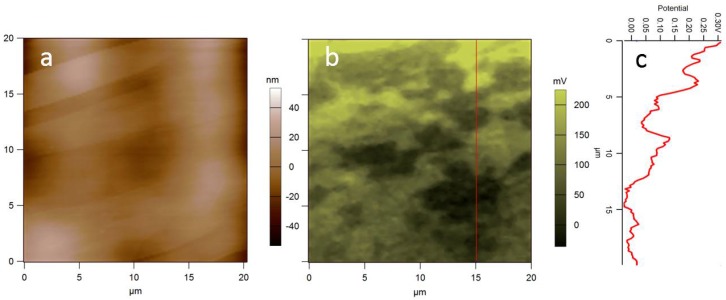
(**a**) Topography and (**b**) surface potential of HOPG, showing both local atomic terraces with different contrast (usually negative, ~30 mV here) and with drifting level during the scan (frame time ~20 min, overall drift ~200 mV). (**c**) Profile of (**b**) at the red vertical line (unpublished data).

In fact, assignment of a well-defined potential level for HOPG is not always straightforward. First, isolated platforms with different potential level often appear in the SKPM images for HOPG, which can probably be ascribed to graphene flakes partly peeling off during cleavage by adhesive tape (see Figure 2b). Additionally, a drift in potential often occurs during imaging HOPG. The level settles down to a constant value after several minutes, a time at least 100-fold longer than that for adsorption of water molecules from the ambient (below one second scale). The effect is probably connected with the orientation of those molecular dipoles (see next section). Whereas the full range of this drift is usually limited to ~100 meV, this issue further limits the accuracy of absolute surface potential values.

However, it should be noticed that, since the tip work function is assumed to be constant over the time and independent of the sample surface being scanned, an important property of Equation (3) can be obtained by taking its local spatial derivatives, which gives:

(5) $$\Delta \Phi_{s}\left(x,y\right)=-e\Delta V_{sp}\left(x,y\right)$$

That is, apart from the coefficient e converting the voltage into energy, the local sample change in work function is equal and opposite to the apparent local change in surface potential. This is the origin of the surface potential contrast in SKPM.

An important issue, especially when trying to evaluate the intrinsic sample property of work function, is the surface contamination of the sample. Dealing with biological samples means taking measurements in a biologically viable ambient, e.g., air as liquid measurement is not possible without extra complications such as tip insulation at its apex and dynamic issues related to the oscillating cantilever. As a consequence of air ambient, pretending to extract reliable information on sample material work function is unrealistic, due to the presence of surface contaminants, from simple water and oxides to organic films [13,14]. Generally speaking, the apparent sample work function when measuring in air is expected to be higher than the real one in vacuum, because of water molecule hydroxyl groups interacting at the tip–sample interface, where the water molecule dipoles are oriented and form a depolarizing medium [15].

The advantage of Equation (5) is that at least the local contrast within a SKPM image can’t be questioned, and this experimental data is valid independent of the source thereof. This is at least valid as long as the tip is unaffected by side-effects due to scanning, such as picking up contamination, which may also change the tip work function.

Seen from another point of view, the issue of surface contamination can be described not in terms of sample modification, but rather as a source of a different electrical field. Indeed, the ∆*V_sp_*(*x*,*y*) contrast in Equation (5) may be due not only to the work function difference on the inhomogeneous sample surface, but also to possible electrostatic charging [8,16,17]. In fact, at any time, build-up of local electrical charge may affect SKPM images, appearing especially at material domain interfaces, but also at morphological edge structures. One possible reason, in addition to the different conductivity of the sample domains, is, e.g., triboelectricity, which can appear after sample treatment prior to imaging but also during imaging itself after occasional contact with the scanning tip. One issue of the static charging effect is that it is generally not controlled and not reproducible, as it can also depend on the temporary relative humidity of the ambient air atmosphere. As such, even the local contrast ∆*V_sp_*(*x*,*y*) should always be confirmed by repeated measurements, and must be assumed as the result of a contingent condition of the tip-sample interface.

### 2.2. More Issues in SKPM Imaging: The Technique

While the Kelvin method for static “probe” position dates back to Lord Kelvin himself [7], its first implementation in a scanning probe microscope was less than 30 years ago [18]. In fact, only in the last 15 years has the method been routinely implemented in commercial AFMs. The physical equations behind the operating principles of SKPM, based on the force between the two plates of a capacitor, can be found elsewhere [1,2,3]. Here we only remind that a feedback loop is set up, which minimizes either the force in amplitude modulation SKPM (AM-SKPM), or the force gradient in frequency modulation SKPM (FM-SKPM) between the two capacitor plates of tip and sample. Under these conditions of zero or constant zero force, Equation (1) is made to hold, and the general principle of the Kelvin probe technique applies.

**Figure 3:**
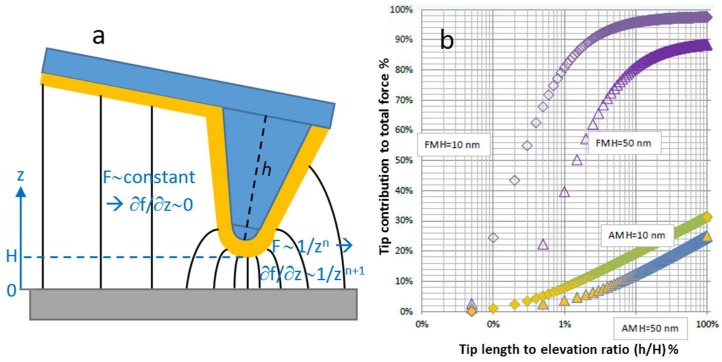
(**a**) Two effects decrease resolution in SKPM vs. AFM: the metal coating (orange) increases the tip apex diameter, and the capacitive coupling between sample and probe (black field lines) extends to both the tip cone and cantilever [21,22,23]. (**b**) In FM-SKPM, the contribution to force by the tip cone is lower than in AM-SKPM (calculations for a SCM-PIT probe, reproduced from Ref. [24] with permission from Bruker Company).

In all cases, the implementation of SKPM suffers from at least one of two issues that critically affect the spatial resolution. One is associated with the tip size. One should remember that a metal-coated AFM tip is typically used. Thus, the thickness of the metal coating sums up—twice—on both sides of the original AFM probe tip, such that if the original tip apex diameter is ~20 nm, and a metal film of typically ~25 nm is overcoated, a total effective tip apex diameter of ~70 nm, results (see Figure 3a). Currently, some manufactures make probes made of a single metal (usually a platinum wire bent at an angle at the free end but also gold is used [19]), such that no metal overlayer is required. Not only does this type of probe limit the tip apex size, but it also addresses successfully the issue of tip scratching, resulting in a loss of conductivity, for the metal overcoated tips. Some researchers also use monolithic semiconductor probes, made of highly doped silicon (resistivity below 0.05 Ω cm).

Generally speaking, the quality of electrical contact is of great importance in SKPM also on the sample side, especially when the sample is a conductor or semiconductor. When it is a dielectric, there is actually not a significant effect in electrical contacting, as in any case its local *V_sp_* is probably determined by the static charge effect, which represents, in this case, the signal to be measured on the sample, rather than an undesired side effect.

Additionally, detrimental to the SKPM image resolution, in one common implementation of SKPM, each line is scanned twice (so-called lift-, nap- or two-pass mode): the first scan tracks the topography in normal mechanical-dithered tapping mode (with main feedback loop circuit), while the second allows to operate the potential feedback loop, during a scan at a given elevation height *H* run at constant distance to the surface features. The reason for this is to decouple the electrical signal from the topographic edges in AM-SKPM, and to avoid convolution with the latter one. This SKPM implementation is similar to electric force microscopy (EFM) and magnetic force microscopy (MFM), except that in those cases, the cantilever is also dithered mechanically instead of electrically (via the AC component of *V_tip_* − *V_sample_*) in the second pass, and without feedback (the signal is qualitative and seen as a shift in cantilever oscillation phase). Given this elevation height, even if sometimes it is very low (a few tens of nm) the lateral resolution is further degraded in AM-SKPM. One fundamental reason is that the electrical force is long range (up to the 10 µm scale) and the probe-sample interaction is capacitive in nature. Thus, because the metal is coated on the entire bottom cantilever side-face, and not only the tip apex, the entire cantilever is the actual capacitor plate opposite to the sample. As a consequence, not only is the resolution degraded, but the measured *V_sp_* is also affected, resulting in typical values of approximately one half of the true ones. This is especially the case when, in samples with regular features, the cantilever hovers over regions with different *V_sp_*, so the effect can sometimes be limited by appropriate cantilever orientation [20].

The limited resolution of the AM-SKPM technique in lift-mode may be minimized by scanning at very low *H*—close to zero, or even below that. In fact, it should be remembered that the *H* is the elevation of the cantilever beam at the bottom position, where the bottom-ward oscillation with −*A* deviation from the cantilever rest position causes the amplitude to match the tapping setpoint (e.g., −40% of amplitude damping with respect to the free amplitude oscillation *A*_0_, meaning that the tip still goes down to −60% *A*_0)_. Thus, it may happen to work with *H* = −20 or −30 nm, and still be able to avoid touching during the second pass (a pre-requisite for this is a flat, featureless sample, such as a 2D material, or in any case a surface with low roughness in the range of 1–50 nm RMS).

As shown qualitatively in Figure 3a, FM-SKPM is less sensitive to the capacitive coupling between sample and probe cone and cantilever, because the force gradient on which the feedback loop is made in this mode changes more steeply with distance from the sample, and thus limits the effective interaction volume to the tip apex only [24] (see also text in Figure 3a). From Figure 3b, it appears that a significant contribution to the capacitive force can be given by the cantilever. This effect is worse for AM-SKPM, where the whole tip cone (with length h) hardly reaches 30%, meaning that 70% comes from the cantilever, whereas for FM-SKPM, the effect is limited, since the tip cone accounts for ~90% of the force, and 50% is determined by the 0.3% bottom-most tip apex.

Another limitation of AM-SKPM in the lift-mode is obviously also the time consumption. The time per frame in this mode is doubled, with respect to a single topographic AFM scan, because each line is scanned twice. Despite these apparent advantages (resolution and faster scan), it appears that some researchers focusing on single molecule imaging still prefer the AM-SKPM, which could apparently provide a better signal-to-noise ratio [25,26,27].

## 3. SKPM Imaging of Biomaterials and Biological Samples

Due to with the restricted field of application of and interest in this review, because biological samples are usually observed in ambient air—if not under liquid medium that is closer to their native conditions—no consideration is given here to SKPM measurements carried out in vacuum or in other artificial inert atmospheres. Actually, for the measurement of intrinsic material properties such as the sample work function, SKPM should be conducted in an inert, oxygen-free atmosphere, e.g., high vacuum or nitrogen saturated glove box [28,29]. Nevertheless, given the nature of AFM as a microscopic imaging technique, most of the presented SKPM examples have taken advantage of patterning to present electrical potential images with contrast within the same scan area, according to Equation (5).

Before reporting about direct SKPM measurements of biomolecules, we present SKPM of the surfaces to be used for living cell cultures, which are lithographically patterned with both additive and subtractive techniques.

### 3.1. Additive Lithography-Manufactured Bio-Substrates

**Figure 4:**
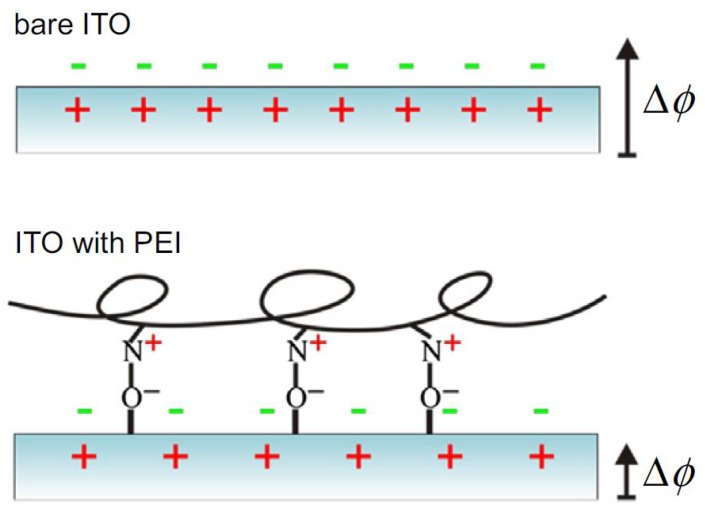
Example of surface charge modification by means of adsorbed molecular layer. In this case, the original charge distribution and existing surface polarization at ITO (top) is modified by the presence of adsorbed PEI. (Reprinted from Ref. [33] with permission; Copyright Elsevier 2015).

Actually, the easiest means to manipulate the electrical potential of a surface to be used in biological applications is to let a molecular layer adsorb to its surface (see e.g. Figure 4). This procedure usually relies on either chemisorption (based on silane chemistry for glass substrates [30] or on thiol chemistry for gold-coated substrates [31,32]), or on physisorption. The latter occurs after simple electrostatic interaction between substrate and adsorbate.

Coating of surfaces with molecular layers, often organic or even biological in nature, has been used, for example, in microelectronic devices, to decrease the work function at the metal and/or semiconductor contact interfaces. The goal is to make it easier to extract electrons from the electrical contacts. The mechanism is that the thin film is chemically or physically adsorbed on the conductor surface, allowing a decrease in its work function by means of the orientation of its molecular dipoles. According to this principle, for example, indium tin oxide (ITO) surfaces have been modified [34] with polyethylenimine (PEI) and similar polymers. Other researchers [34] have also investigated the effect of linear versus branched PEI on ITO contacts, similarly using the surface dipole orientation effect, to model the modification of the work function. The working principle of this method is illustrated in Figure 4. Other materials from the biological field have also been coated on ITO, resulting in a similar decrease in work function, such as, e.g., 3-aminopropyltriethoxysilane [34].

**Figure 5:**
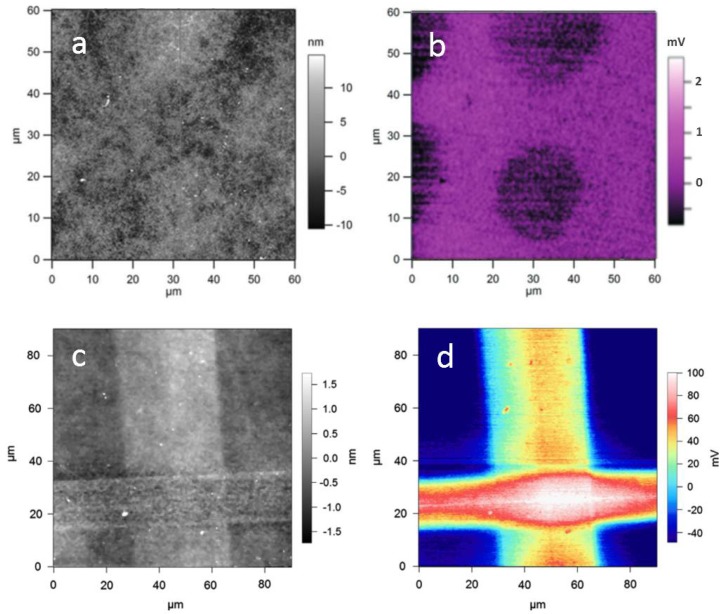
Biomolecular ink spotted onto a substrate by nanoarrayer: (**a**) topography and (**b**) EFM in-phase signal of PDL spots. Reproduced from Ref. [38] with permission; Copyright RSC 2013; (**c**) topography and (**d**) surface potential of a cross-pattern made by two stripes of different molecular ink: PDL (vertical) and PEI (horizontal). Reproduced from Ref. [39] with permission; Copyright Elsevier 2013.

In addition to the possible application of organic films in microelectronic devices, their effect is of major interest as surface coatings for promoting adhesion of living cells, either in view of integration of the substrates into tissues, where they would be the surfaces of biomedical implants, or for fundamental studies with in vitro cell cultures. The reason for this interest is that the electrical properties of the biomaterial surface strongly affect the interaction of living cells adhering to it [35,36]. For example, in [35], the surface potential of polypyrrole–hyaluronic acid (PPy–HA) substrates was investigated under different charging conditions. The surface potential images of uncharged (ground voltage) and charged (+200 mV) PPy–HA showed that the charged films adopted a more uniform potential distribution, with lower and possibly no correlation with the topographical features. In other works, rather than uniformly coating a substrate, a biomolecular ink was deposited selectively to form a pattern, with both chemical and electrically different properties than the surrounding substrate. The goal was to provide living cells in culture to find a patterned substrate surface, to which they could respond differently. For example, in Ref. [37], a square array of round spots of poly-d-lysine (PDL) were deposited by means of direct writing with a nanoarrayer. This patterning was shown to affect not only the mechanical properties of contact stiffness and adhesion, but also the electrical response of the surface, as the PDL molecule is known to be charged positively [38] (see Figure 5b). A quantitative characterization of the electrical potential of patterns made with the same technique yet with different geometry was carried out in Ref. [39]. In that case, two different molecular inks with specific surface charges were used, namely PDL and PEI. The inks were deposited at crossing stripes, and then imaged successfully by SKPM (Figure 5d). It can be seen that the PEI stripe (horizontal) shows higher contrast to the substrate (neutral agarose moist hydrogel) than the PDL stripe (vertical), ~95 vs. ~85 mV, consistent with the expectations, and the effect is cumulative at the crossing point.

### 3.2. Subtractive Lithography-Manufactured Bio-Substrates

One material that has also recently attracted much interest in combination with biological systems is graphene [40,41]. While the many-layer graphene resulting from mechanically exfoliated graphite is mainly used as a filler in functional composites, single-layer graphene (SLG), due to its intrinsically planar (2D) nature, and few-layer graphene (FLG, up to 4 layers) are more promising as substrates for living cell cultures [42,43]. When the intrinsic electrical properties of SLG are of interest, possibly after engineering modifications of the material, true work function values have to be extracted, and are reliable only in the absence of any surface contamination. However, from the point of view of practical applications, the important parameter is again the net surface charge or potential.

As discussed in Section 2.1, the local surface potential contrast is especially clearly interpreted by means of SKPM, and this is also the criterion for controlled adhesion of living cells to SLG coated surfaces. Lorenzoni et al. [11] developed a simple and effective patterning method by pulsed laser ablation of SLG at defined arrays of stripes for the controlled adhesion of primary embryonic hippocampal neurons. After coating the patterned array of SLG onto glass with PDL, the authors observed the growth of an interconnected neuron network mimicking the fabricated pattern. This patterning technique on SLG was also tested with Chinese hamster ovary cells, which, similarly to neurons, showed higher adhesion and consequent initial localization on the SLG-PDL regions, as measured by single cell force spectroscopy AFM [44]. The same group investigated the behavior of neurons on patterned SLG on glass coated with PDL, with immunohistochemical staining and confocal microscopy imaging, as well as electrical transmission at synaptic junctions [45].

**Figure 6:**
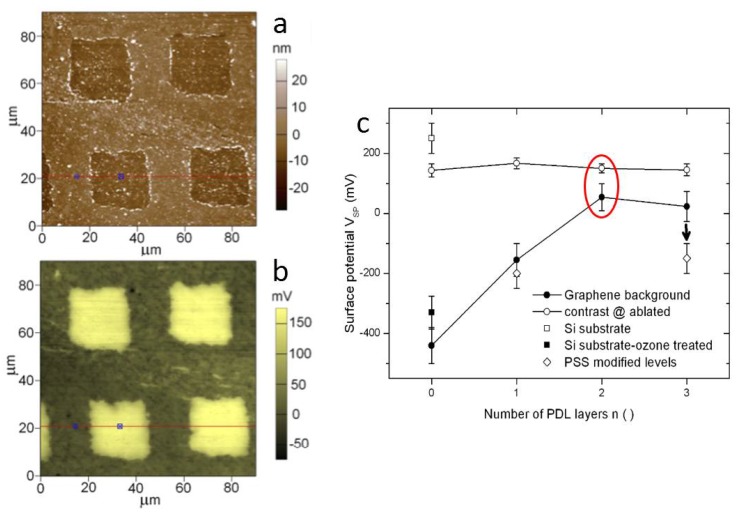
(**a**) Topography and (**b**) surface potential of an array of squares ablated on SLG on glass and coated with PDL (case of two PDL layers). (**c**) Sequence of surface potential values (and contrast of ablated squares versus background) for different numbers of PDL layers, from 0 to 3. The results of adding PSS are also plotted (rearranged from unpublished work [46]).

Given the electrical nature of communication among neuronal cells, as well as the likely electrical contribution to their adhesion to the substrates, SKPM naturally comes into play for substrate characterization. In a series of SKPM measurements, significant electrical potential consequences of the SLG removal for square spots removal was observed on glass. In particular, in Ref. [46] the authors demonstrated the control of the absolute surface potential offset on incremental coating with up to three PDL layers, while the contrast of the ablated square pattern on SLG remained unaffected. The decrease in absolute potential was also demonstrated on adsorption of a polyelectrolyte with negative charge, namely polystyrene sulfonate (PSS), instead of PDL. The exact mechanism of the potential build-up of ablated SLG on glass is yet to be clarified; however, the results have been shown to be reproducible and provide a viable means of spotting adhesion sites for living cells in culture. In Figure 6, an image of a square array of ablated spots on SLG is presented. On silicon (not shown), the contrast at the ablated spots was inverted. The identified means to shift the surface potential may drive and control living cell adhesion also with different types of cells, both electrogenic ones (neurons, cardiomyocytes) and constant potential cells.

### 3.3. A Self-Organized Biosubstrate: Anodic Porous Alumina

Another promising biomaterial [47] to be used as a support for bioassays [48] or biosensors [49,50], which is worthy of being investigated with SKPM, is anodic porous alumina (APA) [51]. The biocompatibility of APA with a variety of living cell types has long been demonstrated [52,53]. APA in itself is chemically and physically inert, but is prone to functionalization by loading or coating the pores with any bioactive agent [54,55]. The pore size and spacing of APA is controlled via the applied voltage during anodization, and can be tuned over a comparatively broad range (5–300 nm) [50]. In addition to the controlled nanoporosity and the function as drug delivery reservoirs, the surface can also be used as a template for in situ positive replication by means of a thin overcoating, e.g., with a gold layer. The latter opens the way to thiol chemistry for further surface modification [56] and makes the APA SERS-active, thanks to the nanoscale size of pores and pore walls [57,58]. This SERS activation is promising in view of either biosensors based on living cells as the sensing element, or bioassays to check the health status of the overcoated cell cultures. In all cases, characterization and possibly control of the electrical surface charge would be of major importance.

So far, the issue of the surface charge of APA has simply been neglected in most cases. Some literature [59] reports that the APA, as fabricated, should carry a negative net electrical charge, due to its oxide nature. Additionally, it should be noticed that anions from the electrolyte are trapped inside the APA walls to a different extent, roughly proportional to the anodizing current (around 5% in sulphuric, oxalic, and phosphoric acid [59]). However, this seems in apparent contrast to the great affinity of APA with all types of living cells, whose membrane also carries a net negative charge. Recent streaming potential measurements of APA [60] has shown that, in disagreement with the expected behavior of aluminum oxide, and supporting the good interaction of APA with living cells, the isoelectric point of APA fabricated in oxalic acid varied between 7.9 (for larger pore diameter of 40 nm) and 6.7 (for pore diameter of 15 nm), as compared to 9 for plain alumina.

**Figure 7:**
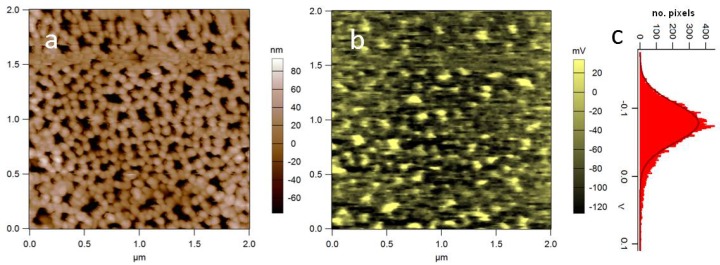
(**a**) Topography and (**b**) SP of APA (acquired in AM-SKPM at *H* = 50 nm); (**c**) distribution of values in (**b**). Cross-talk between height and potential was excluded after observation of flat oscillation amplitude during the nap pass. Significant SP modulation appears, associated with the nanoporous structure (unpublished data).

When we measured APA by SKPM, variable absolute surface potentials were found, according to different anodization conditions and initial aluminum pretreatments. However, in all cases, a local modulation at the pore walls appeared, as a result of the material nanomorphology. In Figure 7, an example of the SKPM image of APA is shown, where a spread appears of more than 60 mV (Figure 7c). We speculate that this modulation may by itself provide a positive cue for living cell adhesion, also given the point that the scale of pore size is comparable with focal adhesion of the adhering living cells. The example of nanoscale surface potential patterning of APA during anodization should be kept in mind as a possible means of easy electrical nanolithography, as this technique is known to apply for several other so-called valve metals, such as Zr, Ta, Nb etc. [61].

### 3.4. Single Biomolecular Constructs

SKPM can be useful not only for the characterization of substrates for biological systems, but also for imaging and to discriminate biological samples of direct interest. In recent years, SKPM has been carried out on proteins [62], DNA [63,64,65] and amyloid fibril aggregates [25,66]. The sensing mechanism of SKPM is promising in this area, as it appears as a label-free detection technique. In the trade-off balance with respect to techniques for measuring in liquid, such as zeta or streaming potential, the disadvantage is the lack of absolute potential measurement in native material condition, while the advantage is the single molecule resolution [67,68].

**Figure 8:**
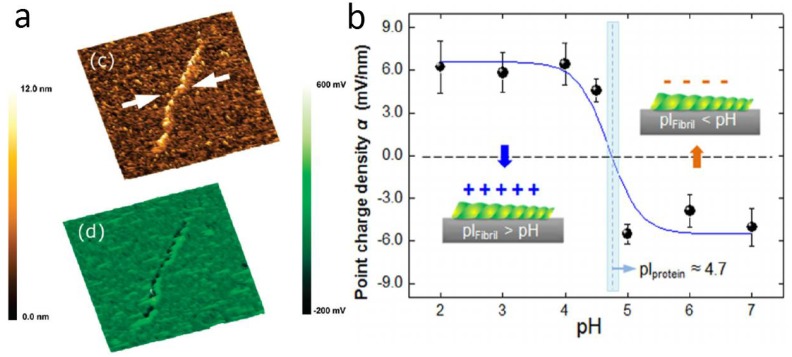
(**a**) SKPM image of single strand DNA (reproduced from Ref. [65] with permission; Copyright ACS 2009); (**b**) apparent surface potential resulting from SKPM images of amyloid fibrils grown in solutions with different pH (reproduced from Ref. [66] with permission; Copyright AIP 2012).

In Ref. [69], the presence of oriented DNA strands was revealed on substrates of Si and glass by using a gold-coated tip modified further with hexadecanethiol. Stretched DNA strands appeared to have higher surface potential with respect to the substrate. In that case, apparently, dipoles formed between the water layer present on the substrate surface and the negatively charged DNA, pointing perpendicular to the surface in the upward direction. However, this experiment could be assigned more to chemical AFM, rather than SKPM, or better to a combination of both techniques, with the tip made specifically for recognizing a given chemical analyte on the sample surface. In Ref. [64], avidin molecules on silicon were observed by SKPM and compared with DNA strands, showing a positive potential contrast with respect to the latter of ~160 mV (see Figure 8a). The measurements were also repeated by the same group on mica substrate [65].

Amyloid fibrils are misfolded protein aggregates associated with a number of neurodegenerative diseases, and their investigation by AFM in terms of mechanical properties has been consolidated [70,71,72]. However, the understanding of fibril formation from amyloid peptides, as well as their interaction with other biomolecules, is still poor, and electrostatic interactions probably play a relevant role in that. In Ref. [73], the surface potential of model lipid membranes was assessed during interaction with Aβ amyloid peptide, and eventually in the presence of 20% cholesterol. The pure lipid layer appeared smooth in surface potential, whereas the lipid with cholesterol showed a potential distribution of 60 mV, which could act as an anchoring pattern for amylogenesis processes. Another group investigated the interaction of amyloid fibrils with gold nanoparticles by EFM [74]. Aβ 25–35 peptides incubated at room temperature for 5–12 days resulted in different fibril morphologies: long according to incubation time for nanoparticle free peptides, and short in bundles with nanoparticles. These morphologies were also associated with different electrical signals. Lee et al. [66] investigated β-lactoglobulin fibrils at the single fibril level, observing different potentials resulting from the pH of the solution used for their preparation (see Figure 8b). The potential changed from +12 to −12 mV when the buffer solution pH changed from 2 to 7, as the isoelectric point pI—i.e., the pH value at which the surface charge is zero—is 5. The morphologies were also correspondingly different, exhibiting protofilaments and more complex, irregular structures, respectively. The observed electrical behavior was not unexpected and points to the necessity of a combined understanding of electrical charge distribution in liquid and solid form after drying. In another work [25], the same group investigated the regrowth of fibrils decomposed by ultrasonic treatment, demonstrating the recurrence of similar potential, concurrently with a corresponding morphology.

At the single-molecule resolution, SKPM has been used also to detect events of specific biomolecular recognition events. For example, in Ref. [26], a sensitive disease-related biomolecular interaction was detected. Tyrosine kinase was activated by adenosine-triphosphate (ATP), while this did not happen when the protein kinases were exposed to a mixture of ATP and its inhibitor Gleevec. In the former case (ATP only), the negative surface potential of the innate protein kinase became more negative than 25 mV, probably due to the negative charges of ATP. However, in the presence of inhibitor, no such change of potential was observed. Thus, SKPM qualifies as a method for possible screening of drugs, testing the efficacy of future nanomedicines.

The use of SKPM as a bioassay for nucleotide analysis was demonstrated in Ref. [75]. A DNA nanoarray was fabricated by dip-pen nanolithography, and SKPM was used to detect hybridization with a complementary DNA functionalized tip. When the DNA matched, the surface potential roughly doubled, from 55 to 110 mV. This case is, however, a combination of SKPM and chemical AFM, rather than an example of a label-free technique.

Similar to the measurement of model lipid layers, SKPM has also been used to measure the potential of living cell membranes, which are known to act with electrical potential modifications, depending on their conditions, and are also affected by the environment. In Ref. [76], the potential of the membrane of PC12 cells was measured, and the effect of different stimulating molecules on that was observed. Despite the wide spread of values under each condition, which may be ascribed to the complex composition of a real cell membrane, the authors were able to identify potential distribution shapes, given by the distribution skewness, associated with the action of the different molecules (H_2_O_2_, dopamine, Zn^2+^).

### 3.5. SKPM of Living Cells

**Figure 9:**
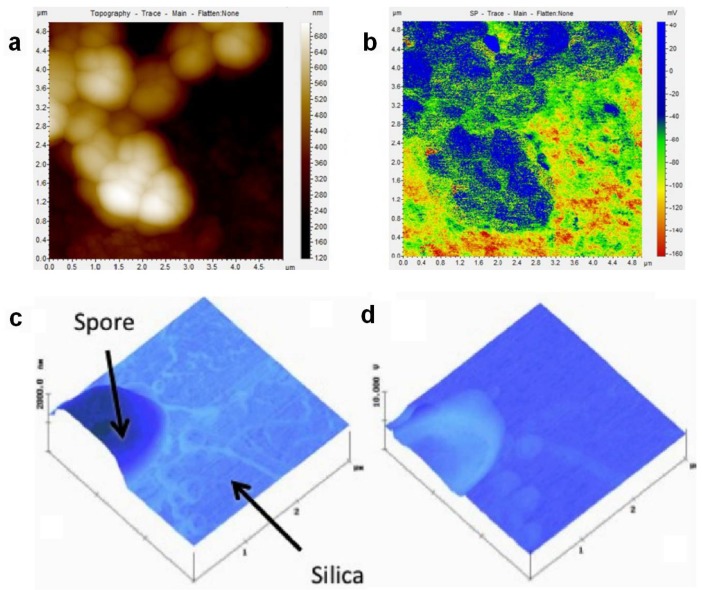
(**a**) Topography and (**b**) SP image of MRSA bacterial cells dried on Poly-L-lysine coated gold (scan size: 4.5 µm). Similar images were obtained on stainless steel, (reprinted from Ref. [77]). (**c**) Topography and (**d**) SP image of dried Bt spore cells on silica (scan size 3 µm, reproduced from Ref. [78]) with permission; Copyright Elsevier 2012).

SKPM in more complex systems such as living cells is even more challenging than on biomolecular layers or sparse biomolecules deposited on well-defined substrates. In fact, to the best of our knowledge, to date it has only been carried out on particularly robust cells such as bacteria [77] and spores [78]. In the former case, Birkenhauer et al. measured the SP of methicillin-resistant *Staphylococcus aureus* (MRSA) cells adhering on stainless steel and gold, providing the procedure also by means of a video. Both types of metal substrate had been functionalized with poly-l-lysine. The edge between cell and substrate was specifically investigated, and, according to the authors, showed changes in SP levels of the cell membrane associated with cellular metabolism and motility. However, it should be noted that the measurements were carried out on dead and dried cells, which may have resulted in quite different SP values than those expected on living cells. Actually, the edge contrast observed could have been due also to topographical artifact, and in fact the authors also noticed that regions with different SP values were observed on both the cells and the cells-free surrounding substrate (see Figure 9a,b).

In Lee et al. [78] the authors investigated the adhesion of spores of *Bacillus thuringiensis* (Bt), which is a species closely related to *Bacillus anthracis*, the cause of anthrax, to solid surfaces of silica, mica and gold (see Figure 9c,d). The measurements were carried out in air, after which the original cast solution containing the spores was dried at different relative humidity (RH) values. It appeared that, as expected for most cells, the SP of the spores was generally more negative than that of the substrates. Actually, the cells were not able to adhere to mica, which has an extremely negative SP. In case of adhesion, the effect of RH was that of a decrease of SP for increasing RH. Despite the apparently reasonable results, it is our personal opinion that, for both mentioned works, the contrast, as well as the SP levels, was probably due to edge effects or topographic cross-talk and to static charge build-up, respectively.

## 4. Conclusions and Perspectives

In the past, SKPM has mainly been used for material science in physics and electronics. However, characterization and control of the electrical surface potential are important also in biological systems and materials, since it is one of the major cues driving the interaction of living cells and tissues with exogenous devices, in, e.g., medical-orthopedics, dental, cardiological, neurological-implants. We pointed to this area of application of SKPM, where the work function of materials is of minor importance, if defined at all, and rather the raw data of apparent potential is of interest.

The surface potential in biosystems is affected by interactions with the environmental medium, as well as by surface contaminants that are always present in biological samples—extracellular matrix, physiological products, residues of advanced chemical functionalization—which make this measurement challenging. We presented examples of SKPM-measured biomaterials, particularly based on adsorption of biomolecular layers and with the function of living cells substrates, because the existing literature on biosamples is rare, especially for living systems. Additionally, because SKPM measurements are carried out in air, extra effort is required to make these results comparable with the information required for liquid operation of biological systems.

Obtaining spatial images of surface potential in physiological conditions would disclose to biological research a visualization of ion-channels or charged molecules embedded in living cell membranes. So far, to the best of our knowledge, only one research group has tried to carry out SKPM measurements in liquid [79], resulting in the measurements presented above for single DNA and protein molecules [64,65].

One of the main drawbacks of doing SKPM in liquid is the requirement that AFM tips be electrically insulated at their apex to prevent current leakage, similar to the probes for electrochemical scanning tunneling microscopy (see, e.g., [80]) or for aperture scanning near-field optical microscopy (see, e.g., [81]). A technique using similar probes and facing similar issues, to be used as a benchmark in this field, is scanning ion conductive microscopy [82].

Another remaining challenge for increased spread of SKPM in the biological area is a proper comparison of the SKPM potential data with those obtained by other techniques specifically engineered for liquid, such as streaming potential for solid surfaces [81] and zeta potential for colloidal particles [82].

When more robust and inexpensive nanoelectrode fabrication, as well as integration of SKPM potential data with the affine electrical techniques, is established, SKPM in biosystems will likely gain a broader diffusion. Hopefully, in the next decade, SKPM will be a valuable tool to investigate the detailed mechanisms of cell transduction and reaction, both on substrates of different nature and after interaction with nanoparticles, either in the surrounding medium or internalized by endocytosis.
